# Deciphering the impact of genetic variants on vulnerability to Opioid Use Disorder

**DOI:** 10.3389/fncel.2026.1814602

**Published:** 2026-09-10

**Authors:** Rajashree Chakraborty, Chittibabu Guda, Avinash Veerappa

**Affiliations:** 1 Department of Genetics, Cell Biology and Anatomy, University of Nebraska Medical Center, Omaha, NE, United States; 2 Department of Genetics, Cell Biology and Anatomy, Center for Biomedical Informatics Research and Innovation, University of Nebraska Medical Center, Omaha, NE, United States

**Keywords:** differential gene expression, exonic variants, genetic variants, long non-coding RNA, nucleus accumbens, Oioid Use Disorder, RNA sequencing, ZNF117

## Abstract

**Introduction:**

Opioid Use Disorder (OUD) is a chronic condition characterized by compulsive opioid intake that drives widespread health, social, and economic burdens.

**Methods:**

To elucidate molecular contributors to addiction susceptibility, we conducted a comprehensive RNA-sequencing analysis of postmortem nucleus accumbens (NAc) tissue from individuals with OUD and matched controls.

**Results:**

Cohort-concordance filtering identified 17 candidate missense variants and one candidate stop-gain variant across 16 genes detected in OUD samples; these RDEVs require DNA-based validation. Missense variants could disrupt key protein domains, and five of these identified variants (*FUT9, FMR1, MFN1, RYR3*, and *DAG1*) have been previously linked to Substance Use Disorders (SUDs) traits. The only stop-gain variant, *ZNF117*, was predicted to produce a truncated protein via impaired folding. Transcriptomic profiling followed by Ingenuity Pathway Analysis (IPA) predicted activation of neurodevelopmental programs, with 145 upregulated and 29 downregulated genes collectively implicating pathways related to synaptic plasticity, neuronal differentiation, and axon guidance. Importantly, differential expression of long non-coding RNAs (lncRNAs), including *LINC01554* and *LINC00996*, was identified, with putative regulatory associations with key transcription factors (TFs) such as *NPAS4* and *GADD45B*.

**Discussion:**

Together, these findings provide an integrated view of candidate genetic and transcriptomic alterations in OUD and identify lncRNA-centered regulatory networks and candidate variant-bearing genes as hypothesis-generating leads for future functional and DNA-based validation studies.

## 1 Introduction

OUD is a chronic condition marked by the persistent use of opioids that results in significant socio-economic distress or impairment. Opioids encompass natural, semi-synthetic, and fully synthetic drugs, ranging from prescription pain medications (e.g., oxycodone or morphine) to illicit substances such as heroin. Globally, OUD affects over 16 million people, with approximately 2.1 million cases reported in the United States alone (Dydyk et al., 2026). The progression from substance use to addiction occurs along a continuum-from initial physical dependence to full-blown addiction-following broadly a three-stage addiction (US Department of Health and Human Services, 2016). In the binge/intoxication stage, dopaminergic projections from the Ventral Tegmental Area (VTA) to the NAc mediate reward learning and habit formation; in the withdrawal/negative effect stage, stress-related neuroadaptations occur within the extended amygdala to drive dysphoria and anxiety; and in the preoccupation/anticipation stage, dysregulated prefrontal cortical circuits, along with insular and hippocampal contributions, underlie intense craving and impaired executive control (Koob and Volkow, 2016). During the binge/intoxication stage, the NAc mediates drug rewards via dopaminergic signaling and is linked to anhedonia and cue-induced craving (US Department of Health and Human Services, 2016; Koob and Volkow, 2016; Goldstein and Volkow, 2011).

Neurodevelopmental and neurofunctional alterations underlying the addiction cycle are further shaped by genetic influences, which significantly contribute to both the risk of OUD and individual variability in treatment response, as supported by twin studies and large-scale genomic analyses demonstrating a strong heritable component across substances (Freda et al., 2021; Hatoum et al., 2023). Research by Tsuang et al. (2001) underscores this genetic influence, suggesting that genetic markers could help identify susceptible individuals while also highlighting an interplay with the psychosocial environment (Na et al., 2024; Freiermuth et al., 2023). For example, genetic variants within the “reward” and opioid metabolism pathways have significant positive (*DRD3* and *CYP3A5*) and negative (*CYP3A4* and *CYP1A2*) associations with OUD (Freiermuth et al., 2023). Complementing these findings, GWAS in opioid users have identified key OUD-associated variants associated with persistent users, with co-occurring genes implicating several addiction-related pathways (Panday et al., 2024). Further reinforcing the genetic basis of OUD, a cross-ancestry meta-analysis of 425,944 individuals identified 14 OUD-associated loci (12 of which were novel), including variants in *OPRM1* and *FURIN*, and revealed significant genetic correlations with 127 OUD-associated traits (Kember et al., 2022). Genetic variants have been shown to significantly influence gene expression in humans, contributing to phenotypic diversity and disease susceptibility (Hulse and Cai, 2013). Genetic variants in OUD modulate gene expression patterns, thereby shaping individual susceptibility to the disorder and influencing treatment outcomes (Chundru et al., 2023; Gaddis and Mathur R, 2022).

Although several OUD-associated genetic traits have been identified, a clear mechanistic understanding of how DNA variation influences downstream transcriptomic alterations in key reward-related brain regions, such as the NAc, remains lacking. To date, only one RNA-sequencing (RNA-seq) study has explored genetic variants in the ventral midbrain of postmortem opioid-exposed individuals (Ajmeriya et al., 2025); however, no comparable study has focused on the NAc, a region as critically involved in the brain's reward circuitry. Furthermore, the specific genetic variants driving maladaptive gene expression changes in NAc during the progression from opioid exposure to dependence is yet to be elucidated. To address this gap, our study performs joint variant discovery and transcriptome profiling on human NAc RNA-seq samples, directly illuminating how genetic variants shape gene regulation in OUD and identifying candidate variants that confer functional susceptibility at the molecular level.

**Figure 1:**
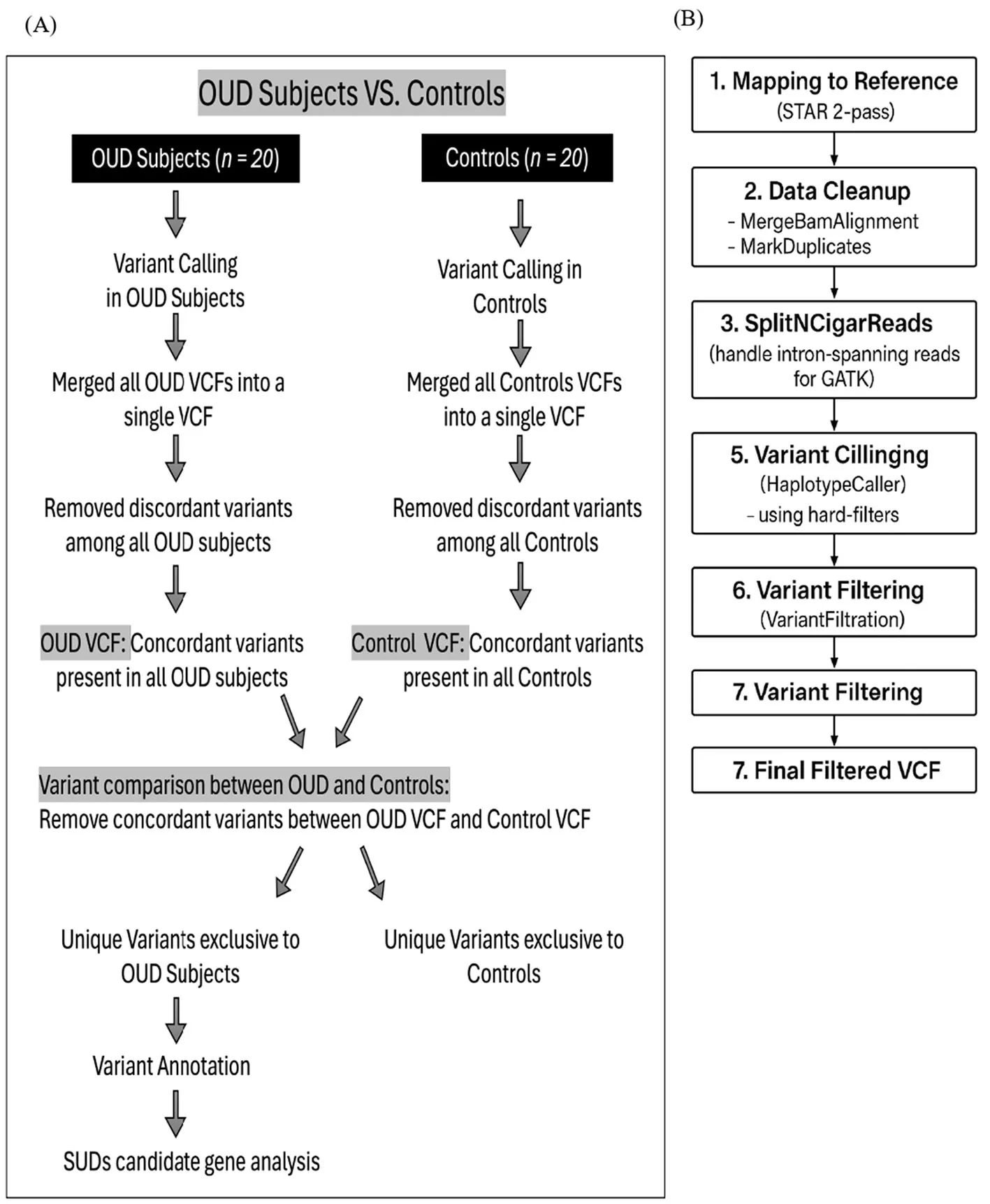
Schematic Diagram for detecting Opioid Use Disorder (OUD)-specific exonic variants. **(A)** shows data analysis steps begining from RNA sequencing (RNA-seq) data from 20 OUD and 20 non-user nucleus accumbens (NAc) samples were processed into individual Variant Call Format (VCF) files, merged by cohort, and filtered to retain RDEV present in all OUD subjects but absent in non-users. **(B)** shows the pipeline steps involved in processing RNA-saq data.

Due to the limited availability of paired whole genome or exome and transcriptome datasets from OUD-associated cohorts, we leveraged RNA sequencing (RNA-seq) data for calling variants to investigate the association between the variants and gene expression linked to OUD. Here, we analyzed raw FastQ reads from human NAc tissue samples of opioid users and non-users with the following objectives: (a) to identify RDEV and differentially expressed genes (DEGs) associated with substance use and addiction; (b) to correlate these variants with specific SUDs traits; and (c) to elucidate the relationship between these variants and DEGs contributing to transcriptomic dysregulation in OUD. Our study identified damaging and deleterious variants (Figure 1) in genes involved in multiple stages of the addiction cycle within the NAc region. Notably, we uncovered 18 candidate RDEVs (17 missense, 1 stop-gain) in 16 genes detected in OUD samples under our cohort-concordance filter, including a stop-gain in ZNF117 that we modeled structurally. We emphasize that many candidates have high reported population allele frequencies, and all require DNA-based validation. Several missense changes from these RDEVs mapped to critical protein domains showed correlations with SUDs traits. By integrating these variant calls with expression analyses, we identified dysregulated SUD-linked genes whose expression patterns correlate with the discovered RDEVs. This study also revealed putative lncRNA-mRNA regulatory networks that may mediate regulatory mechanisms. Together, these findings provide an integrated view of candidate genetic and transcriptomic alterations in the NAc, generating hypotheses for future DNA-based validation and functional studies.

## 2 Methods

### 2.1 Sample collection and pre-processing

The study (Seney et al., 2021) cohort comprised 40 post-mortem subjects divided equally into two groups: 20 individuals who met diagnostic criteria for OUD at the time of death and 20 unaffected non-user subjects matched appropriately based on sex and age. The OUD group had a minimum illness duration of 5 years (range: 5–18 years since diagnosis), and both groups were excluded for any history of major psychiatric or neurological disorder (other than OUD in the case group), significant head injury, or evidence of neurodegenerative disease. Mean age did not differ between groups (47.3 ± 9.5 years in non-users vs. 46.9 ± 7.3 years in OUD), and each group comprised 10 males and 10 females. Racial composition was 13 White and 7 Black individuals in the unaffected comparison group vs. 19 White and 1 Black individual in the OUD group.

The NAc region was identified from fresh-frozen coronal tissue blocks of the right hemisphere using established anatomical landmarks by the original contributors (Seney et al., 2021). Approximately 50 mg of tissue was collected using a cryostat-based dissection method designed to minimize contamination from adjacent white matter and other striatal subregions, while preserving RNA integrity. Key tissue quality and metadata metrics were comparable between OUD and non-user cohorts. The average postmortem interval (PMI) was 15.7 ± 6.1 h for OUD samples and 16.0 ± 5.3 h for controls. Brain pH values were 6.6 ± 0.3 and 6.4 ± 0.2, and RNA integrity numbers (RIN) averaged 8.0 ± 0.7 and 7.8 ± 0.7 for OUD and control groups, respectively. The mean time of death, measured in zeitgeber time (ZT), was 6.16 ± 6.28 for OUD and 7.72 ± 7.42 for controls. Tissue storage time prior to processing averaged 100.3 ± 86.1 months for OUD samples and 103.0 ± 59.7 months for controls (Seney et al., 2021).

Paired-end RNA-seq data in FastQ format for the NAc cohort were retrieved from the Sequence Read Archive under accession PRJNA739548 (Seney et al., 2021), encompassing both OUD samples and matched healthy non-users. Metadata and raw paired-end FastQ files were retrieved using the nf-core/fetchngs pipeline (v1.3) on Nextflow (v21.04.0) (Ewels et al., 2020). A list of study sample IDs was passed to the pipeline, which translated them into ENA-compatible experiment identifiers via the ENA API; the API also supplied associated metadata and direct URLs to download FastQ files. All files were fetched in parallel with curl, and MD5 checksums were computed to confirm file integrity.

### 2.2 RNA-seq quantification

Gene expression quantification was performed within the *Nextflow* framework (v21.04.0) using the *nf-core/rnaseq* pipeline. Briefly, raw sequencing reads underwent initial quality assessment with *FastQC* (v0.11.9) to confirm the absence of adapter contamination and overrepresented sequences. Random 5′ nucleotides were trimmed using the *extract* function in *UMI-tools*, followed by removal of residual Illumina adapter sequences (13 bp; AGATCGGAAGAGC) using *Trim Galore!* (v0.6.6). Ribosomal RNA reads were subsequently filtered with *SortMeRNA* (v4.3.4). Cleaned reads were aligned to the GRCh38 reference genome in two-pass mode using *STAR* (v2.6.1d), and resulting BAM files were sorted and indexed with *SAMtools* (v1.12). PCR duplicates were collapsed using the *dedup* function in *UMI-tools*, followed by *Picard MarkDuplicates* (v2.23.9) to identify residual library duplicates. Transcript assembly was performed with *StringTie* (v2.1.7) for quality control purposes only and was not used for downstream differential expression analysis. Transcript- and gene-level abundance estimates were generated using *Salmon* (v1.4.0) against GENCODE v38 annotations, and these quantifications served as the primary input for downstream differential expression analyses. Post-alignment quality control metrics, including coverage uniformity, strand specificity, GC and nucleotide biases, duplication rates, and sequencing saturation, were evaluated using *RSeQC* (v3.0.1), *Qualimap* (v2.2.2-dev), *dupRadar* (v1.18.0), and *Preseq* (v3.1.1), with all reports consolidated using *MultiQC* (v1.9).

### 2.3 Differential gene expression analysis

Salmon transcript-level abundance estimates were imported into R (v4.2.1) using tximport (Bioconductor v3.16) and summarized to gene-level counts. Lowly expressed genes (counts per million < 1 in fewer than two samples) were filtered out. All differential expression analysis was performed in DESeq2 (v1.36.0) using the design formula ~ age + sex + race + PMI + RIN + condition, with condition (OUD vs. non-user) as the variable of interest. DESe2′s median-of-ratios method was used for normalization, negative binomial generalized linear models were fit, and the Wald test was applied with empirical Bayes shrinkage of dispersion estimates. *P*-values were adjusted using the Benjamini-Hochberg false discovery rate procedure, and genes with adjusted *p* < 0.05 and |log_2_ fold change| >1 were considered significant. We note that cell-type composition was not estimated or modeled; this limitation is addressed in the Discussion.

### 2.4 Variant discovery and annotation filtering strategy

The cohort-concordance filter applied here is a direct methodological extension of the framework published by Veerappa and Guda (2024), where the identical vcffilterjdk concordance operator was used to retain variants present in all affected probands and absent in family member controls across five SUD pedigrees. We apply this framework under the same descriptive, hypothesis-generating logic, extended from a pedigree-based cohort to an unrelated case-control RNA-seq cohort (*n* = 40). At *n* = 20 per group, formal variant-level enrichment testing would be substantially underpowered for the rare-variant signals that are the focus of this work; the cohort-concordance filter is therefore applied as a candidate-identification approach rather than as a statistical association test. The filter is a deterministic selection operator that maps the full variant call set to the subset of variants exhibiting perfect partitioning between cohorts, analogous to co-segregation analysis in Mendelian disease genetics (Jarvik and Browning, 2016; Biesecker et al., 2024), where co-segregation of a variant with affected status across all affected individuals and its absence in all unaffected individuals constitutes the canonical evidentiary pattern (Veerappa and Guda, 2024; Jarvik and Browning, 2016; Biesecker et al., 2024). We emphasize, however, that this analogy is procedural, not evidentiary. Unlike Mendelian disorders, in which such co-segregation patterns can provide strong evidence for pathogenicity, OUD is a highly polygenic complex trait, and we therefore use this approach solely to nominate candidate genes, not as evidence that these are causal OUD risk variants. The resulting variant list is therefore presented as a candidate set requiring orthogonal DNA-based validation, rather than as a list of statistically associated loci. The criterion is intrinsically conservative: detection in all 20 OUD subjects and absence in all 20 non-user subjects represents one of the most stringent partitioning criteria definable on a binary call matrix. For a variant whose true detection probability is independent of cohort assignment, the probability of perfect partitioning by chance is bounded by *p*^20^ × (1–*p*)^20^, on the order of 10^−12^ even at the maximally adverse *p* = 0.5, placing the filter output far below conventional significance thresholds without requiring a separately computed empirical *p*-value. This bound assumes detection probability independence between cohorts, an assumption that may be violated where transcript expression or coverage differs systematically between OUD and non-user samples-a possibility we address through explicit AF-based caveats below. Several important caveats apply to this approach. First, detection is restricted to expressed exonic regions; variants in unexpressed, lowly expressed, or non-coding regions are not detectable. Second, RNA-seq cannot reliably distinguish germline from somatic variants, and DNA-based sequencing is required to make this distinction. Third, RNA-seq is susceptible to artifacts from RNA editing; although we filtered A > G substitutions at known editing sites using curated databases, novel or rare editing events may not be fully excluded. Fourth, absence of a variant call in a sample may reflect insufficient read coverage or low transcript expression rather than true genomic absence; the cohort filter is therefore sensitive to systematic expression or coverage differences between groups. Fifth, the filter is particularly susceptible to ambiguity for variants with high reported population allele frequencies; where such variants meet the filter criteria, their absence in non-users could reflect either true cohort-specific presence or RNA-seq detection failure. We flag candidates with AF > 0.8 in Table 1 and restrict primary biological interpretation to lower-AF candidates pending DNA-based validation. Throughout this manuscript, we therefore describe these variants as RNA-seq–detected expressed exonic variants (RDEVs) in the OUD cohort rather than as germline OUD-vulnerability variants. Whole-genome or whole-exome sequencing in an independent cohort is required to establish any of these RDEVs as germline susceptibility variants. We considered assigning empirical significance to this filter by permuting case-control labels; we do not adopt this approach because label permutation assumes sample exchangeability, which does not hold for RNA-seq variant calls where detection depends on condition-dependent transcript coverage (evidenced by the 174 DEGs reported here). Under these conditions a permutation-derived null conflates coverage-driven detection asymmetry with random variation and does not yield an interpretable significance value; indeed, recovery of qualifying variants under permuted labels would itself confirm that perfect partitioning is not evidence of disease association, consistent with our treatment of these variants as candidates rather than associated loci.

**Table 1:** List of gene variants identified in this study.

Variant location	Variant	Amino acid change	Variant_type	Gene_symbol	SIFT	PolyPhen	AF
chr2:97194750	T>C	p.L825S	Missense	ANKRD36	Deleterious (0.04)	Benign (0.066)	-
chr2:97204194	A>T	p.T998S	Missense	ANKRD36	Deleterious (0.03)	Benign (0.053)	-
chr3:49510575	C>G	p.S14W	Missense	DAG1	Deleterious_ low_confidence (0.03)	Benign (0)	0.9659
chr4:55858412	G>A	p.G30E	Missense	EXOC1	Deleterious_low_confidence (0)	Possibly_damaging (0.888)	-
chr3:58156064	A>G	p.M2293V	Missense	FLNB	Deleterious_low_confidence (0.01)	-	-
chrX:147943227	G>A	p.E458K	Missense	FMR1	Deleterious_low_confidence (0)	-	-
chr6:96203864	A>G	p.T237A	Missense	FUT9	Deleterious (0.02)	Benign (0.007)	0.8984
chr14:31395279	C>G	p.P173A	Missense	HEATR5A	Deleterious_low_confidence (0.01)	Benign (0.09)	0.8287
chr5:55941690	G>A	p.E717K	Missense	IL6ST	Deleterious (0)	-	-
chr3:179375226	A>G	p.I328V	Missense	MFN1	Deleterious (0.01)	Probably_damaging (0.957)	-
chr14:49828721	G>A	p.G440E	Missense	NEMF	Deleterious_low_confidence (0.02)	Probably_damaging (0.997)	-
chr14:49832244	A>T	p.S257C	Missense	NEMF	Deleterious_low_confidence (0.01)	Probably_damaging (0.977)	0.9273
chr1:96769865	G>A	p.G93E	Missense	PTBP2	Deleterious_low_confidence (0)	Benign (0.215)	-
chr15:33662451	C>T	p.R1641C	Missense	RYR3	-	Possibly_damaging (0.577)	0.8383
chr3:129827886	A>G	p.S165G	Missense	TMCC1	Deleterious_low_confidenc e(0)	Benign (0.011)	0.9653
chr2:178580041	G>C	p.A22416P	Missense	TTN	-	-	0.9946
chr7:64978289	C>T	p.R428^*^	Stop_gain	ZNF117	-	-	0.8818
chr2:218454934	T>C	p.L979S	Missense	USP37	Deleterious_low_confidence (0)	Benign (0.335)	0.9838

This table presents the genomic position, type of variant and amino acid change, impacted gene, and predicted Sorting Intolerant From Tolerant (SIFT) and Polymorphism Phenotyping (PolyPhen) scores.

### 2.5 AlphaFold2 modeling

**Figure 2:**
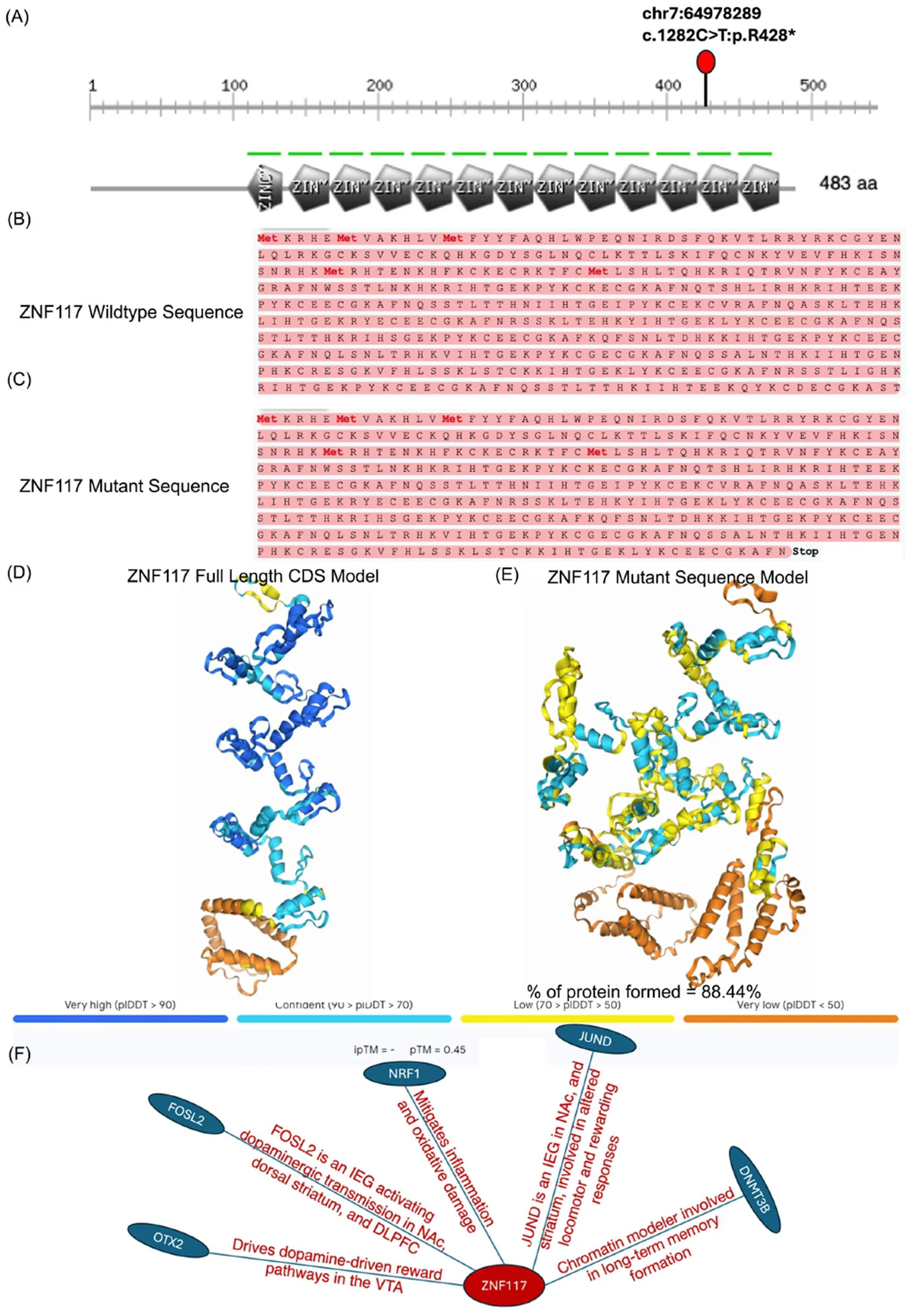
**(A)** Nonsense variant in Zinc Finger Protein 117 (ZNF117) (p. Arg428Ter) and predicted protein structures. Schematic of the ZNF117 gene show chromosome 7:84,978,289 (c.1282C>T; p.R428*). **(B)** Wild-type ZNF117 sequence. **(C)** Mutant ZNF117 sequence highlighting the premature stop codon. **(D)** Three-dimensional (3D) model of the full-length ZNF117 protein (color scale indicates model confidence). **(E)** Predicted truncated protein structure, indicating approximately 88.4% of the full-length sequence is retained. **(F)** shows upstream regulators of ZNF117 along with their functional roles.

Wild-type ZNF117 (full-length residues 1–483) and the truncated p.R428^*^ variant (residues 1–427) were modeled using the ColabFold implementation of AlphaFold2 (v2.3.0) via the Google Colab notebook server (sokrypton/ColabFold). Multiple sequence alignments were generated with MMseqs2 against the UniRef and environmental sequence databases. Predictions employed the “monomer” model preset with five model ensembles, three recycling iterations, and default tolerance thresholds, followed by *in silico* Amber relaxation of each model; all other parameters were set to default (Jumper et al., 2021; Mirdita et al., 2022). Per-residue confidence was assessed using the predicted Local Distance Difference Test (pLDDT) score, represented in Figure 2 by the following color scheme: dark blue (>90, very high confidence), cyan (70–90, confident), yellow (50–70, lower confidence), and orange ( ≤ 50, very low confidence). This enabled direct comparison of the C-terminal zinc-finger architecture between the wild-type and p.R428^*^ models and evaluation of local confidence differences between the two structures.

### 2.6 Missense variant mapping and domain annotation

For each missense variant, we first retrieved the corresponding RefSeq transcript and its protein translation in FASTA format from NCBI. Variant positions were mapped by converting genomic coordinates to transcript offsets, then the single-nucleotide change was introduced *in silico* at the codon level to generate mutant DNA and amino acid sequences. Both wild-type and variant protein sequences were then submitted to NCBI's Conserved Domain Database via the CD-Search web service, using the default RPS-BLAST parameters (*e*-value cutoff = 0.01; composition-based statistics enabled). Domain hits were filtered at the default score thresholds and their boundary coordinates compared between the two sequences. By overlaying the variant position onto the annotated domain architecture, we identified any shifts in domain boundaries or complete loss of functional motifs attributable to the amino acid substitution (Yang et al., 2020).

### 2.7 lncRNA-mRNA interaction prediction and regulatory network analysis

Dysregulated lncRNAs and mRNAs with a log_2_fc |1| with *p* ≤ 0.05 were paired and submitted to lncRRIsearch (seed length = 7 nt; maximum 200 target sites, energy cutoff = −16 kcal/mol) and RNAhybrid (default seed region = 7 nt, no bulge constraints, allowing G: U pairing reporting the lowest-energy hybrid) for interaction prediction. Only transcript pairs detected by both tools were retained as high-confidence lncRNA-mRNA interactions. These pairs were then used to construct a regulatory network, and the corresponding binding energies were assembled into a matrix for hierarchical clustering (euclidean distance, and Ward's linkage) in R to delineate core lncRNA-mRNA regulatory modules (Fukunaga et al., 2019).

### 2.8 Integrated pathway analysis

In our integrated pathway analysis (IPA), differentially expressed gene lists (FDR ≤ 0.05 and log_2_FC > |1|) were imported into Ingenuity Pathway Analysis (QIAGEN) and subjected to the Core Analysis pipeline to identify enriched canonical pathways, upstream regulators, and network interactions based on curated literature and public data sets. Briefly, gene identifiers with associated expression values were uploaded using default mapping parameters, and Core Analysis was run with a right-tailed Fisher's exact test to calculate pathway enrichment p-values, which were then adjusted using the Benjamini-Hochberg method with a significance cut-off of *p* < 0.01. Visualization of top pathways and networks was performed within IPA's graphical interface, and results were exported as tables and network figures for further interpretation (Krämer et al., 2014).

## 3 Results

### 3.1 Identification of cohort-concordance filtered RDEVs

RNA-seq reads were generated from NAc tissue in postmortem samples from individuals with OUD (*n* = 20) and age- and sex- matched non-users (*n* = 20). As described in the Methods (Figure 1), individual VCF files were compressed, indexed, and merged to produce two aggregate files: one specific to the OUD cohort and one for non-users. Within the OUD group, only RDEVs that appeared consistently in all individuals were retained. Comparing this “common OUD variant” set with the merged non-user variant set identified a cluster of RDEVs that were universally present in the OUD cohort yet entirely absent in non-users (Supplementary Table 1). We filtered out all frameshift variants consisting of T or A insertions to mitigate potential false positives arising from RNA A-to-I editing events (Mai and Chuang, 2019). Several candidate RDEVs in Table 1 have high reported population allele frequencies (AF > 0.8), indicating they are common in the general population. Their apparent absence in our non-user samples could reflect either true biological cohort-specificity or RNA-seq detection limitations-a question that cannot be resolved without DNA-based sequencing in the same individuals, which we identify as a priority for follow-up validation. These high-AF candidates are therefore reported for transparency but should not be interpreted as cohort-specific without DNA validation; subsequent mechanistic interpretation focuses primarily on lower-AF candidates, where the detection-bias concern is reduced though not eliminated. Across the candidate set, most missense variants were predicted to have damaging or deleterious effects on protein function (Supplementary Table 1), and the stop-gain in ZNF117 is predicted to produce a truncated protein. Several variant genes are linked to diverse cellular functions - including cytoskeletal organization (FLNB), membrane trafficking (EXOC1), and transcription/translation control (FMR1, PTBP2). Their roles in opioid use remain to be established, and the candidate RDEVs identified here represent hypothesis-generating findings that require DNA-based confirmation and functional validation in follow-up studies.

### 3.2 Characterization of RDEV impact on protein domains

A total of eighteen single-nucleotide RDEVs were identified across sixteen genes, each predicted to change critical structural or functional domains (Table 2). Two variants in *ANKRD36* localize to an intrinsically disordered region (*PTZ00121*), indicating potential disruption of conformational flexibility or protein-protein interactions. A variant in *DAG1* affects the signal-peptide segment of dystroglycan-1, which may impair proper precursor processing. In *EXOC1*, the alteration maps to the pleckstrin-homology domain, suggesting altered membrane association of the exocyst complex. The *FLNB* variant resides at the INPPL1-binding interface of filamin-B, while the *FMR1* change targets a disordered region essential for RANBP9-mediated nuclear export. In *FUT9*, the variant occupies the donor-binding pocket of its glycosyltransferase fold. The *HEATR5A* variant falls within a HEAT-repeat domain, and the *IL6ST* alteration maps to a disordered segment of the interleukin-6 receptor β-subunit. The *MFN1* variant impacts the dynamin-like GTPase domain, and two independent variants in *NEMF* affect disordered regions of the ribosome-quality control complex. The *PTBP2* variant lies within the RNA-binding PTB domain, and the *RYR3* change localizes to its characteristic repeat units. A *TMCC1* variant is positioned in a coiled-coil/disordered region, whereas variants in *TTN* and *ZNF117* disrupt a fibronectin type III domain and a C2H2 zinc-finger motif, respectively. Finally, the *USP37* variant resides within the ubiquitin carboxyl-terminal hydrolase domain, implicating a possible effect on deubiquitinating activity. Collectively, these findings point to potential disruptions in protein stability, trafficking, and interactions, warranting further functional validation (Table 2).

**Table 2:** Summary of variant genes and their affected protein domains.

Gene	Variant	Protein domains affected
ANKRD36	T>C	Disordered structure, PTZ00121
ANKRD36	A>T	Disordered structure, PTZ00121
DAG1	C>G	Dystroglycan 1 preproprotein, Sig-Peptide AA:
EXOC1	G>A	PH-EXOC1, exocyst complex component 1 isoform 1
FLNB	A>G	Filamin-B isoform 2, Interaction with INPPL1
FMR1	G>A	Fragile X messenger ribonucleoprotein 1 isoform ISO1, Interaction with RANBP9, Disordered, Required for nuclear export
FUT9	A>G	4-galactosyl-N-acetylglucosaminide 3-alpha-L-fucosyltransferase 9, Glycosyltransferase family 10, donor-binding
HEATR5A	C>G	HEAT repeat-containing protein 5A
IL6ST	G>A	Interleukin-6 receptor subunit beta isoform 1 precursor, interleukin-6 receptor subunit beta isoform 1, disordered
MFN1	A>G	Mitofusin-1, Dynamin-like protein including dynamins, mitofusins, and guanylate-binding proteins
NEMF	G>A	Ribosome quality control complex subunit NEMF isoform 1, ribosome rescue protein RqcH, archaeal type, disordered
NEMF	A>T	Ribosome quality control complex subunit NEMF isoform 1, ribosome rescue protein RqcH, archaeal type, disordered
PTBP2	G>A	Polypyrimidine tract-binding protein 2 isoform 6, hnRNP-L_PTB
RYR3	C>T	4 X approximate repeats, ryanodine receptor 3 isoform 1
TMCC1	A>G	Transmembrane and coiled-coil domains protein 1 isoform a, disordered
TTN	G>C	Titin isoform IC, Fibronectin type 3 domain
ZNF117	C>T	COG5048, C2H2 Zn finger
USP37	T>C	Ubiquitin carboxyl-terminal hydrolase 37

This table highlights the genes harboring missense or stop-gained variants, along with the protein domains or structural/functional motifs potentially affected by these variants. The domain annotations are derived from established protein family databases (e.g., Pfam, InterPro) and literature reports.

### 3.3 Association of RDEVs with substance use disorders

From the 17 missense RDEVs identified, we highlighted five specific missense variants (*FUT9, FMR1, MFN1, RYR3*, and *DAG1*) that have prior literature support. The *FUT9* variant shows findings where *FUT9*-deficient animals indicate elevated anxiety-like behavior linked to altered neural connectivity. *FMR1* variants are known to disrupt synaptic plasticity and reward-impulse control pathways, aligning with risk factors for binge eating. *MFN1*, a key mediator of mitochondrial fusion, has been tied to calcium-dependent synaptic activity and implicated in reward-processing adaptations. *RYR3* variants affect neuronal excitability, influencing locomotor activity, social interactions, and learning mechanisms. Finally, *DAG1* plays a recognized role in synapse formation and maintenance, particularly at inhibitory connections, and is critical for proper synaptic development and function (Table 3).

**Table 3:** Phenotypic implications of identified missense gene variants.

Gene	Function	Associated behavior	Mechanism
**FUT9**	Glycosylation, neural cell adhesion	Anxiety	Alters neural connectivity and receptor signaling
**FMR1**	Regulation of neural plasticity, impulse control	Binge eating (risk factor for addiction)	Influences reward circuitry and impulse control
**MFN1**	Mitochondrial fusion, calcium signaling	Reward processing, addiction maintenance	Regulates calcium-dependent synaptic activity
**RYR3**	Intracellular calcium regulation, synaptic plasticity	Locomotor activity, social behavior	Affects neuronal excitability and learning processes
**DAG1**	Synapse formation, nerve-glia interactions	Memory, synaptic plasticity	Modulates synapse formation and neuronal interactions

Summary of key missense variant genes with known neural functions, associated behaviors, and proposed molecular mechanisms.

### 3.4 Functional analysis of a stop-gain variant in ZNF117

*ZNF117*, a zinc-finger protein, was the only gene found to harbor a stop-gain variant within the identified RDEVs. This protein plays a role in DNA-TF binding, zinc-ion binding, and the regulation of DNA-templated transcription. To investigate the structural consequence of a stop-gain variant in *ZNF117*, we analyzed a C>T transition at chromosome 7:64,978,289 (c.1282C>T; p.R428^*^), which introduces a premature stop codon (Figure 2A). This variant truncates the wild-type 483-amino-acid *ZNF117* protein at position 428, leading to the loss of the two C-terminal zinc-finger domains. Sequence alignment of the wild-type and mutant proteins predicted a truncation of the final 55 amino acids in the mutant isoform (Figures 2B, C). Structural predictive modeling with AlphaFold further demonstrated a protein configuration change resulting from the stop-gain variant (Figures 2D, E). The truncated form retains approximately 88.44% of the original protein sequence but lacks key structural regions associated with zinc-finger motifs, which may be critical for DNA binding and transcriptional regulation. Notably, transcriptional network analysis revealed that *ZNF117* acts as a downstream target of several key regulators, including *FOSL2, JUND, NR1F1*, and *OTUD2* (Figure 2F). These TFs are implicated in immediate early gene activation, chromatin remodeling, and dopaminergic signaling, suggesting that truncation of *ZNF117* may disrupt neurobiological pathways relevant to neurodevelopment, reward processing, and memory formation.

### 3.5 Differentially expressed genes and pathway analysis in OUD vs. non-users

**Figure 3:**
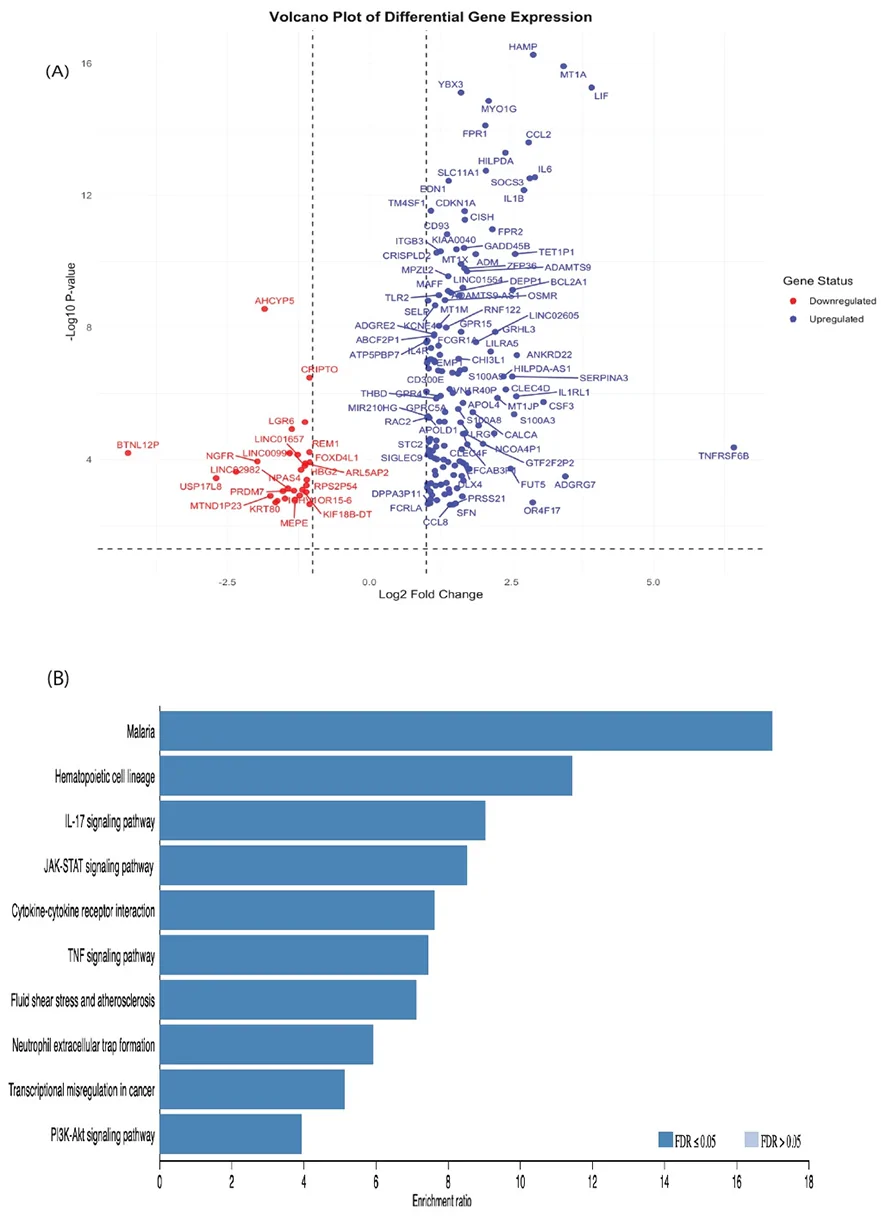
**(A)** Identification of differentially expressed genes (DEGs) in Opioid Use Disorder (OUD) cohorts. Each point represents a gene labeled by its symbol, with log2 fold change on the x-axis and negative log10 *p*-value (–log10 *p*-value) on the y-axis. Red indicates significantly downregulated genes, and blue indicates significantly upregulated genes (*p* < 0.05). **(B)** Top enriched pathways among upregulated DEGs at false discovery rate (FDR) <0.05.

Subsequently, we analyzed DEGs between OUD and non-user cohorts with a filter of log2FC, which quantified the change in gene expression on a base-2 logarithmic scale. A threshold of log2FC > 1 or log2FC < −1 and a *p* value < 0.05 was applied to identify genes exhibiting significant upregulation or downregulation, respectively. The resulting DEGs between the two cohorts are presented in Figure 3A and Supplementary Table 2. Notably, we identified several genes including *IL1B, IL1R, IL4R, IL6, CP, CSF3, EDN1, EMP1, GPR4, GRHL3, HAMP, HILPDA, KCNE4, KCNJ15, MAFF, MPZL2, MYO1G, NGFR, SELP, SERPINA3, SIGLEC9, SLC11A1, SLC2A5, SOCS3, ZFP36*, and *DLX4*, which were previously attributed with SUDs.

We further performed enrichment analysis to identify top enriched pathways (FDR < 0.05) among the upregulated DEGs identified in our analysis (Figure 3B). This observation underscores the critical role of immune-related mechanisms in our dataset. Furthermore, several other enriched pathways - including those related to immune signaling-suggest a coordinated regulation of host defense and inflammatory responses. Collectively, these findings imply that dysregulated immune function may be associated with the observed transcriptional alterations and offer avenues for further mechanistic investigation. We note that bulk RNA-seq cannot distinguish whether the observed immune pathway enrichment reflects per-cell transcriptional upregulation or relative expansion of immune-related cell populations (e.g., microglia) in OUD tissue. Supplementary Figure 1 illustrates the top enriched pathways linked to the downregulated DEGs. Although these pathways had an FDR > 0.05, these results point to a potential suppression of specific biological processes, including those involved in immune regulation and other essential cellular functions. In addition, several additional enriched pathways highlight a coordinated downregulation of key signaling networks. Taken together, these observations indicate the potential interplay of regulatory mechanisms and provide probable targets for future mechanistic and therapeutic studies. Please note, in the pathway enrichment analyses (Figure 3B, Supplementary Figure 1), the apparent significance of the “malaria” pathway among upregulated genes and the “virion” pathway among downregulated genes reflects shared gene crosstalk inherent to pathway-based analyses. These enrichments are primarily driven by overlapping immune and inflammatory gene sets, rather than indicating true biological links to malaria or viral infection. This underscores that the observed pathway enrichment is a result of shared immune-related gene sets, not evidence of actual infection-specific processes.

**Figure 4:**
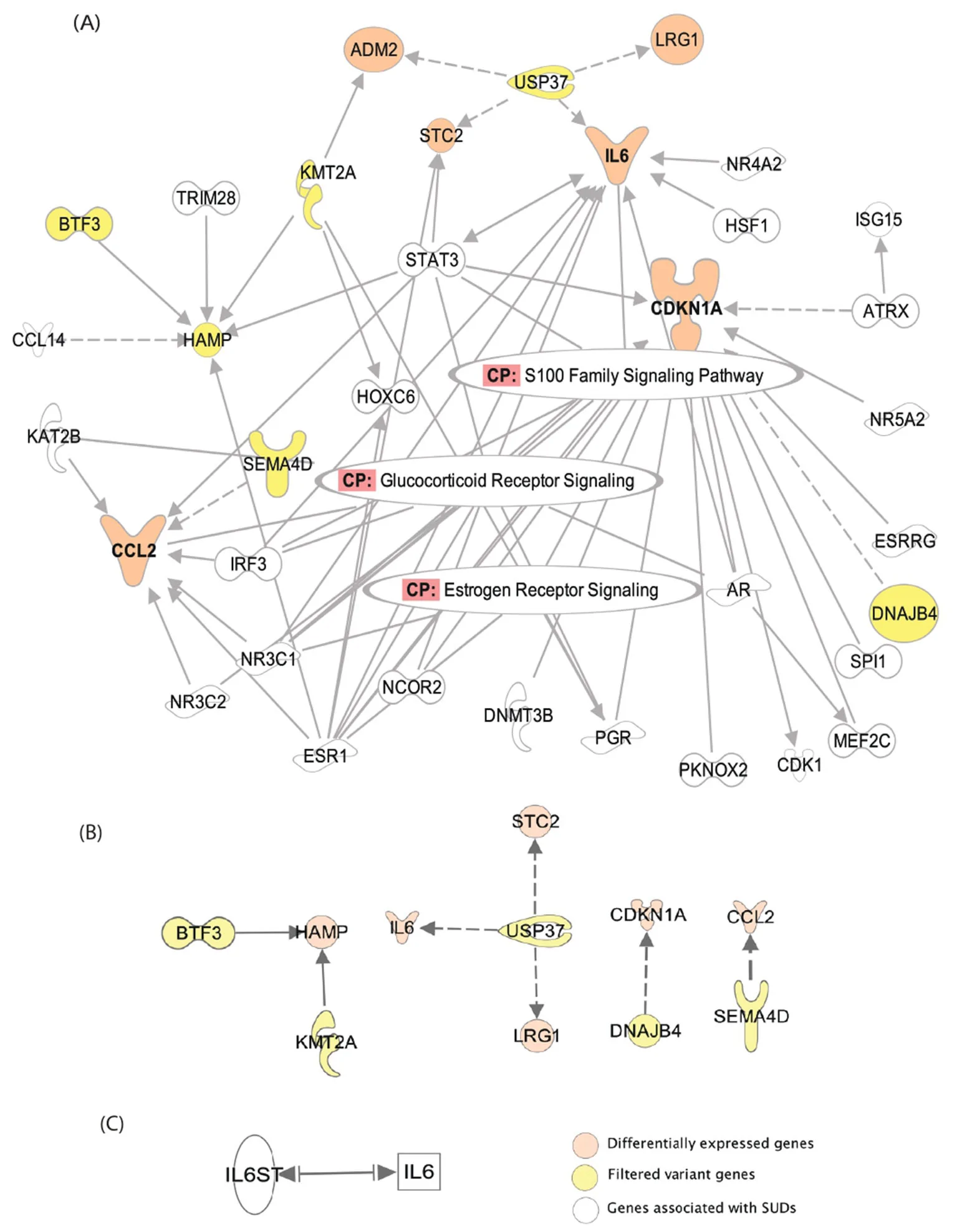
**(A)** Network visualization showing DEGs, genes carrying filtered variants, and genes associated with SUDs). Edges represent known or predicted interactions, and key canonical pathways (S100 family signaling, glucocorticoid receptor signaling, and estrogen receptor signaling) are highlighted. **(B)** Magnified view of selected interactions between DEGs and genes carrying filtered variants. **(C)** Simplified schematic of the IL6/IL6ST regulatory axis.

To assess the direct impact of the identified RDEVs on DEGs, we next evaluated potential regulatory interactions in IPA between the identified variants and differentially expressed genes (Figures 4A–C). Our analysis did not reveal any direct interactions, except for the *IL6* and *IL-6ST* pair (Figure 4C), which are known to bind and initiate signal transduction. Subsequent network analyses did uncover both direct and indirect interactions between various gene variants and DEGs (Figure 4B). Moreover, we observed interactions linking variant genes, DEGs, and established SUDs-related genes, along with the activation of S100, glucocorticoid, and estrogen signaling pathways (Figure 4A).

### 3.6 lncRNA-mRNA regulatory networks underlying SUDs

**Figure 5:**
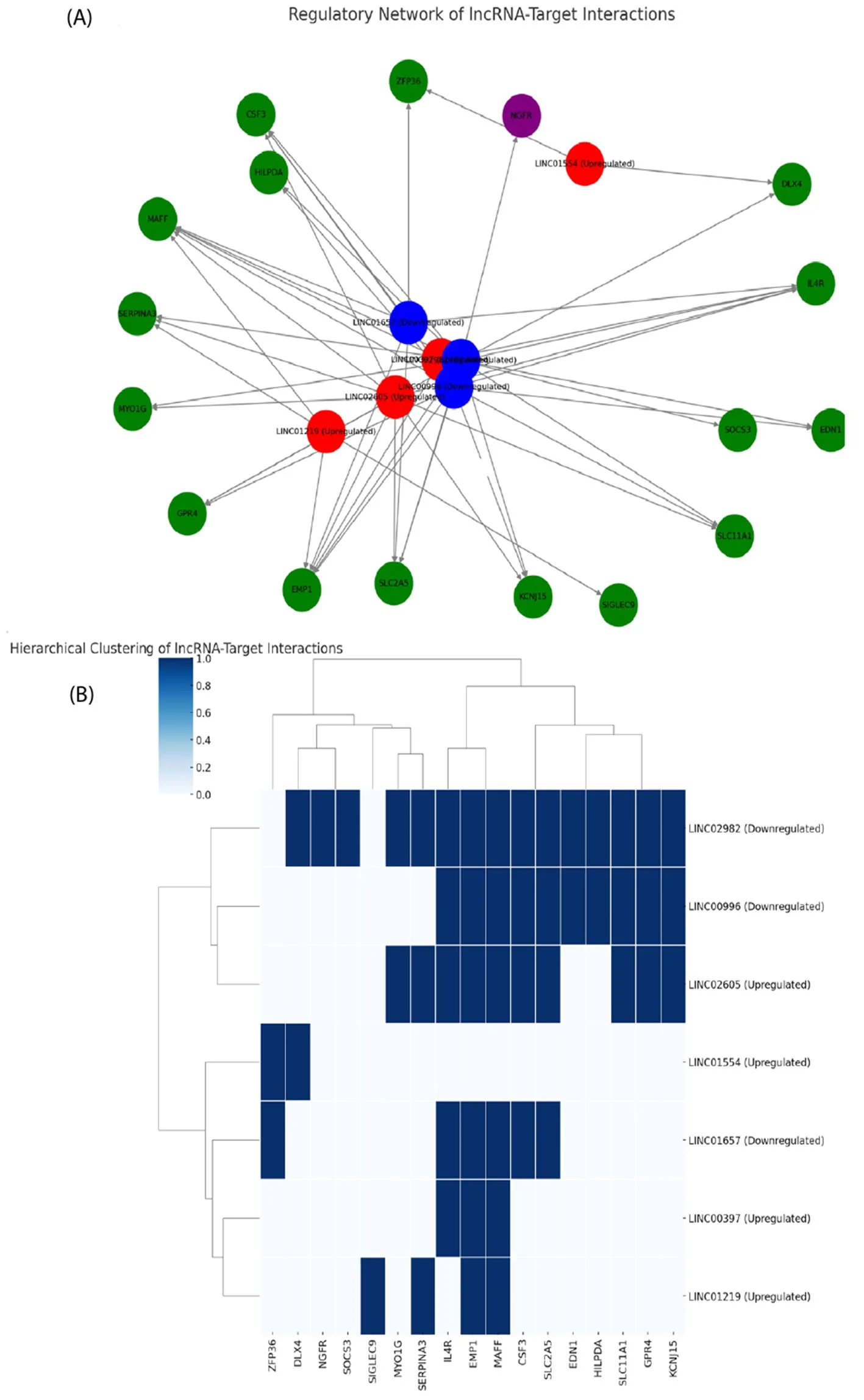
**(A)** Network of all remaining long non-coding RNA messenger RNA (lncRNA-mRNA) target interactions. **(B)** Hierarchical clustering of these lncRNA-mRNA interactions.

Following DEG analysis, we next investigated the relationship between the differentially expressed mRNAs and differentially expressed lncRNAs to explore potential interactions between the two transcript classes (Figures 5A, B). We predicted regulatory relationships between long non-coding RNAs (lncRNAs) and their target mRNAs based on hierarchical clustering analyses (Table 4). In the positive correlations (Table 4A), upregulation of lncRNAs such as *LINC00397, LINC01219, LINC01554*, and *LINC02605* was associated with increased expression of several core genes including *SERPINA3, SIGLEC9, ZFP36*, and *DLX4*, among others. Conversely, inverse correlations (Table 4B), were observed where downregulation of lncRNAs like *LINC00996, LINC01657*, and *LINC02982* corresponded to increased expression of several targets (e.g.*, CSF3, EDN1, GPR4, HILPDA, KCNJ15, MYO1G, SERPINA3, SLC11A1, SLC2A5, SOCS3*, and *DLX4*), with a notable exception for *NGFR*, which showed reduced expression when *LINC02982* was downregulated, suggesting a potential inverse co-regulatory relationship (Table 4). Several core genes (e.g., *IL4R, MAFF, EMP1*) were found in both positive and inverse correlations, suggesting context-dependent regulation. Further differential expression analysis of lncRNAs revealed significant changes within the cohort, with upregulation of *LINC01554* and *GADD45B*, and downregulation of *LINC00996* and *NPAS4*. (Supplementary Figures 5A–D). Notably, interactions between *LINC01554* and *NPAS4* as well as between *LINC00996* and *GADD45B* were identified, and both *NPAS4* and *GADD45B* have been previously implicated in SUDs (Supplementary Figures 5E–H). Clustering analysis of lncRNAs indicated that *LINC02982* and *LINC00996* (both downregulated) share similar regulatory patterns, suggesting they may co-regulate overlapping pathways, while *LINC02605* (upregulated) is predicted to form its own regulatory cluster (Figure 5). Additionally, *LINC01657* (downregulated) and LINC00397 (upregulated) exhibit some common regulatory influences. At the gene level, *IL4R, CSF3, EMP1*, and *MAFF* emerged as core regulatory hubs, whereas *MYO1G, NGFR*, and *SOCS3* formed a distinct subgroup, and *ZFP36, DLX4*, and *SIGLEC9* clustered together, supporting a shared regulatory role among these genes (Figure 5). Collectively, these results forecasted a complex network of lncRNA-mediated regulation that may contribute to the transcriptional changes observed in SUDs, highlighting potential regulatory hubs and signaling pathways for further mechanistic investigation.

**Table 4:** Correlations between long non-coding RNAs (lncRNAs) and their target genes.

lncRNA	Target genes
**(A) Positive correlations (when lncRNA is upregulated, target is also upregulated)**.	
**LINC00397**	IL4R, EMP1, MAFF
**LINC01219**	EMP1, MAFF, SERPINA3, SIGLEC9
**LINC01554**	ZFP36, DLX4
**LINC02605**	IL4R, CSF3, EMP1, GPR4, KCNJ15, MAFF, MYO1G, SERPINA3, SLC11A1, SLC2A5
**(B) Inverse correlations (when lncRNA is downregulated, target increases)**.	
**LINC00996**	IL4R, CSF3, EDN1, EMP1, GPR4, HILPDA, KCNJ15, MAFF, SLC11A1, SLC2A5
**LINC01657**	IL4R, CSF3, EMP1, MAFF, SLC2A5, ZFP36
**LINC02982**	IL4R, CSF3, EDN1, EMP1, GPR4, HILPDA, KCNJ15, MAFF, MYO1G, SERPINA3, SLC11A1, SLC2A5, SOCS3, DLX4
**LINC02982**	NGFR

Long Non-Coding RNAs (lncRNAs) expression changes correlate with changes in target gene expression. In (A), upregulation of each lncRNA corresponds to concurrent upregulation of the listed target genes. In (B), downregulation of each lncRNA corresponds to an increase in the listed targets, suggesting potential inverse regulatory relationships.

## 4 Discussion

In this study, we identified eighteen variants that are consistently present in the NAc of individuals with OUD yet entirely absent in matched non-users. Sixteen genes harboring these 18 exonic changes, *ANKRD36, DAG1, EXOC1, FLNB, FMR1, FUT9, HEATR5A, IL6ST, MFN1, NEMF, PTBP2, RYR3, TMCC1, TTN, ZNF117* and *USP37*, were identified and annotated. All but one stop-gain variant were missense variants predicted to moderately affect protein function and addiction-relevant neural signaling pathways. We then examined the affected domains and considered the phenotypic implications of these variants in the context of SUDs. Next, we characterized how the stop-gain variant influences *ZNF117* protein formation. We also analyzed DEGs and explored their interactions with both the variant genes and SUD-associated genes. Finally, we detected lncRNA expression among the DEGs and further investigated their regulatory interactions.

### 4.1 RDEVs and their potential impact on protein function in OUD subjects

The identification of 18 candidate RDEVs under our cohort-concordance filter highlights several pathways that may merit further investigation in relation to opioid vulnerability (Table 1). Variants in *FLNB*, an actin-crosslinking protein that stabilizes F-actin networks and scaffolds membrane receptors during neuronal migration and synaptogenesis, suggest disrupted cytoskeletal integrity may impair synaptic structure and plasticity (Table 2) (Sheen et al., 2002; Sutherland-Smith, 2011). Changes in *EXOC1*, a core component of the exocyst complex responsible for tethering post-Golgi vesicles and delivering AMPA receptors to the postsynaptic membrane, imply dysregulated receptor trafficking and synaptic plasticity (Gerges et al., 2006). Finally, variants in *RYR3*, the ryanodine receptor that amplifies intracellular Ca^2+^ signals via calcium-induced calcium release, highlight potential perturbations in Ca^2+^-dependent pathways fundamental to reward-related synaptic remodeling and addictive behaviors (Li et al., 2018). These candidate RDEVs implicate cytoskeletal scaffolds, vesicular-tethering machinery, and calcium-signaling modules as candidate areas for functional follow-up in opioid pathogenesis, contingent on DNA-based confirmation. Mapping of OUD-specific variants onto both intrinsically disordered regions and conserved structural modules suggests multiple modes of functional disruption. Two missense variants in *ANKRD36* and *IL6ST* localize to intrinsically disordered segments, which often undergo disorder-to-order transitions to mediate transient, regulatory protein-protein interactions (Chakrabarti and Chakravarty, 2022). The stop-gain in *ZNF117* truncates two C2H2 zinc-finger motifs, likely abolishing DNA-binding specificity and transcriptional regulation (Li et al., 2022). Together, these findings suggest that these RDEVs might contribute to OUD-related neuroadaptations, even though the literature linking these specific genes to addiction remains sparse. These results are therefore warranting follow-up validation in relevant neural models.

### 4.2 Association of RDEV genes with the SUDs-associated phenotype

Among the candidate RDEVs, several reside in genes with prior literature support for substance use phenotypes. We note that FUT9, RYR3, and DAG1 carry high reported population allele frequencies; the gene-level association with SUDs is established in prior literature, but the specific variants identified here in these genes require DNA-based confirmation before they can be interpreted as OUD-associated. Subsequently, we investigated whether the 16 genes harboring missense variants were associated with any SUDs-associated phenotype (Table 3). We observed that *FUT9* plays a critical role in neural cell adhesion (Gouveia et al., 2012), thereby affecting neural connectivity and receptor signaling, and has been associated with anxiety (Abdullah et al., 2022). The *FMR1* gene, which regulates neural plasticity, impulse control, and the reward circuitry, has been linked to binge eating-a potential indicator of addiction vulnerability (Blanco-Gandia et al., 2021; Kirkpatrick et al., 2016). *MFN1*, essential for mitochondrial fusion (Liu et al., 2023) and the regulation of calcium-dependent synaptic activity (Pyakurel et al., 2015), has been implicated in reward processing and the maintenance of addiction (Jiang et al., 2024). Additionally, *RYR3*, involved in neuronal excitability and learning processes (Liu et al., 2014; Tedoldi et al., 2020) was found associated with disruptions in locomotor activity and social behavior (Matsuo et al., 2009). Lastly, *DAG1*, which modulates synapse formation and neuronal interactions (Nickolls and Bönnemann, 2018; Jahncke and Wright, 2023), has been connected to addiction-associated synaptic plasticity. Overall, these gene-phenotype associations underscore the need for further investigation into how these variants collectively shape diverse addiction-related traits and inform targeted interventions.

All five missense variant genes linked to SUD traits are also key to neurodevelopment. *FUT9* synthesizes the Lewis X epitope to regulate neural stem cell proliferation and differentiation via Notch signaling; its loss disrupts cortical development and emotional behavior (Yagi et al., 2012). *FMR1* encodes FMRP, essential for synaptic protein synthesis and dendritic spine maturation; its absence impairs synaptic plasticity and cognitive function (Mercaldo et al., 2009). *MFN1* supports mitochondrial fusion during neural induction; its deficiency leads to mitochondrial fragmentation, oxidative stress, and impaired neuronal differentiation (Knott et al., 2008). *RYR3* mediates Ca^2+^-dependent hippocampal plasticity, with variants linked to infantile spasms and epilepsy (Balschun et al., 1999). *DAG1* maintains neural architecture and myelination by organizing extracellular matrix, guiding migration, and supporting inhibitory synapses (Sciandra et al., 2023).

### 4.3 Functional implications and downstream interactions of stop-gain RDEV ZNF117

Before discussing the predicted structural and regulatory consequences of the ZNF117 stop-gain, we note that this candidate variant has a high reported population allele frequency (AF ≈ 0.88). Its apparent absence in our non-user samples maybe explained by RNA-seq detection limitations. The structural and regulatory analyses below should be interpreted as predicted consequences of the truncation if the variant is confirmed in OUD samples by DNA-based sequencing, not as established OUD-associated findings. DNA validation is identified as the priority next step for this candidate. The premature stop codon in *ZNF117* (Figure 2) is predicted to excise the two C-terminal zinc-finger motifs that are essential for its DNA-binding and transcriptional regulatory functions (Nabeel-Shah et al., 2024). This truncation predicted a functionally impaired isoform of *ZNF117* that is incapable of engaging its normal gene targets and may also trigger nonsense-mediated decay (Rehwinkel et al., 2005). *ZNF117* is expressed in the basal ganglia, including the NAc, confirming its presence in the relevant brain circuitry (Sjöstedt et al., 2020). Functional evidence from glioblastoma stem cell studies demonstrates that *ZNF117* regulates differentiation via the Notch signaling pathway, linking it to neural cell fate mechanisms underlying neuroplasticity and potentially relevant to reward-related adaptations (Liu et al., 2022). Given that *ZNF117* sits downstream of immediate-early factors like FOSL2 and JUND, which coordinate chromatin remodeling and dopaminergic signaling, the loss of its transcriptional activity could derail networks that underlie reward learning and memory consolidation (Chottekalapanda et al., 2020). Such disruption of *ZNF117*-mediated regulatory circuits in the NAc may therefore contribute to the maladaptive neuroplasticity observed in OUD.

### 4.4 OUD specific RDEVs affect neurodevelopmental regulators

One of the key things we observed is that the OUD-specific RDEVs identified in this study map to core neurodevelopmental regulators. For example, *FMR1* controls dendritic spine maturation and mGluR5-dependent synaptic plasticity in hippocampal neurons (Zhang et al., 2009; Levenga et al., 2011); *PTBP2*, a splicing factor is essential for neuronal maturation, axonogenesis, and cortical circuit formation (Li et al., 2014); and *IL6ST* (gp130), whose JAK/STAT signaling drives radial glia proliferation and proper cortical layering (Heinrich et al., 1998). Structural genes such as *DAG1* and *FLNB* guide neuronal migration and cytoskeletal integrity, while MFN1-mediated mitochondrial fusion meets the bioenergetic demands of progenitor differentiation (Yamada et al., 2018). The premature stop-gain in *ZNF117* likely abolishes its C2H2 zinc-finger-mediated transcriptional repression, disrupting progenitor proliferation and differentiation and thereby heightening neurodevelopmental susceptibility to opioid dependence (Al-Naama et al., 2020). The strength of this prediction depends on DNA-based validation given the high reported population allele frequency of this variant.

### 4.5 Differential gene expression and immune dysregulation in OUD

Our DEG analysis revealed 145 upregulated and 29 downregulated genes (Figure 3A, Supplementary Table 2). Notably, several upregulated genes such as *IL1B, IL6, IL4R, SOCS3* are well-documented mediators of immune and inflammatory responses (Croker et al., 2012; Yoshimura et al., 2012; Wei et al., 2020). Particularly, the pronounced enrichment of immune system (Figure 3B) processes–illustrated by the prominent immune–related pathway enrichment verifies that chronic substance use provokes a sustained inflammatory response (Morcuende et al., 2021). Conversely, the downregulated DEGs (Supplementary Figure 1), despite showing an FDR of >0.05, indicate a suppression of specific biological processes, including pathways integral to immune regulation and other cellular functions (Kohno et al., 2019). The downregulation of these pathways suggests that, alongside an overactive immune response, there might be a concurrent loss of essential regulatory mechanisms. This duality-enhanced inflammatory signaling paired with impaired regulatory control, may contribute to the neurobiological complexity underlying SUDs, warranting further mechanistic studies to disentangle these relationships.

Further, the activation of additional pathways, such as cytokine signaling (Ahearn et al., 2021), glucocorticoid (Srinivasan et al., 2013), S100 Family, and estrogen signaling (Ahmed et al., 2024), underscores a complex regulatory network that could modulate immune mechanisms (Figures 4A–C). This aligns with existing research indicating that immune dysregulation is a hallmark of many neuropsychiatric conditions, including SUDs (Loftis and Huckans, 2013). While our targeted evaluation of potential interactions between identified genetic variants and DEGs did not reveal extensive direct interactions, the exception of the *IL6* and *IL-6ST* pair is noteworthy (Figure 4C). This finding is of importance given the established role of *IL6/IL-6ST* signaling in mediating inflammatory responses (Yao et al., 2014) and cellular stress (Darcy and Tseng, 2020). Furthermore, our broader network analyses uncovered both direct and indirect interactions among variant genes, DEGs, and previously established SUDs-associated genes (Figure 4A). The complex interplay among these molecules, especially through key signaling networks, highlights potential regulatory nodes that may serve as targets for therapeutic intervention.

Preclinical studies consistently show that attenuation of neuroinflammatory signaling, particularly via glial modulators like ibudilast, reduces opioid reward, tolerance, and withdrawal symptoms (e.g., hyperalgesia and pain behaviors), suggesting a direct modulatory role of immune pathways in addiction-related phenotypes (Metz et al., 2017; Toloff and Woodcock, 2022; Butelman et al., 2023). Reviews further demonstrate that opioids, including morphine, fentanyl, methadone, buprenorphine, and oxycodone directly modulate immune cells (macrophages, T-cells, NK cells) and alter cytokine profiles via both central neuroimmune and peripheral mechanisms (Franchi et al., 2019). Moreover, opioids can activate toll-like receptor 4, triggering release of pro-inflammatory cytokines such as *IL-1*β and *TNF-*α, mechanisms implicated in the development of opioid tolerance, hyperalgesia, and addiction behaviors (Eidson et al., 2017). However, we note that whether these signals reflect upregulated immune signaling within individual cells or shifts in cell-type composition (particularly microglial expansion) cannot be resolved from bulk RNA-seq and warrants single-nucleus follow-up.

### 4.6 lncRNA-mRNA regulatory networks in the context of SUDs

This study presents lncRNA-mRNA analysis (Table 4) that delineates a complex landscape of lncRNA-mediated regulation in SUDs as previous reported (Saad et al., 2019; Seyednejad and Sartor, 2022; Denham et al., 2022; Farris and Mayfield, 2021). Our hierarchical clustering analyses (Figures 5A, B) revealed that the upregulation of lncRNAs-including *LINC00397, LINC01219, LINC01554*, and *LINC02605*-is associated with increased expression of key genes such as *IL4R, EMP1, MAFF, SERPINA3, SIGLEC9, ZFP36*, and *DLX4*. This positive association suggests that these lncRNAs may act as enhancers of gene expression within pathways critical for immune signaling and cellular stress responses (Mathy and Chen, 2017; Kim et al., 2015). In contrast, we observed inverse relationships where the downregulation of lncRNAs like *LINC00996, LINC01657*, and *LINC02982* corresponded to the upregulation of multiple target genes including *IL4R, CSF3* among others, with the notable exception of *NGFR*, which showed reduced expression levels. This pattern hints at a potential co-regulatory suppressor function for certain lncRNAs (Huang et al., 2012; Wang and Chang, 2011), thereby adding another layer of complexity to the transcriptional regulation in SUDs. Further differential expression analysis underscored the significance of these regulatory elements, revealing that lncRNAs such as *LINC01554* and *GADD45B* were upregulated, while *LINC00996* and *NPAS4* were downregulated (Supplementary Figures 2A–H). The observed interactions between *LINC01554* and *NPAS4*, as well as between *LINC00996* and *GADD45B*-both of which have been previously implicated in SUDs (Zipperly et al., 2021; Sosnowski et al., 2022)-suggest that these lncRNAs might be intricately involved in regulation of the molecular pathways underlying addiction vulnerability (Seyednejad and Sartor, 2022). At the gene level, the identification of *IL4R, CSF3, EMP1, and MAFF* as core regulatory hubs further emphasizes the central role of these genes in the transcriptional network in SUDs (Wei et al., 2023; Friske et al., 2025; Randesi et al., 2017). Moreover, the formation of distinct subgroups, such as the clustering of *MYO1G, NGFR*, and *SOCS3* (Andersen et al., 2015; Malewska-Kasprzak et al., 2024; Zhang et al., 2021) vs. *ZFP36* and *DLX4* (Zhu et al., 2018; Lynch et al., 2008) supports the existence of coordinated regulatory modules that may influence different aspects of SUD pathophysiology (Figure 5). Collectively, our findings support the hypothesis that lncRNAs are key modulators of gene expression in SUDs, potentially orchestrating the dysregulation of immune-related and other signaling pathways.

The study made use of a rare, well-characterized cohort of postmortem NAc tissue from individuals with OUD, enabling integration of RDEV discovery and transcriptomic analysis in a brain region central to addiction. While based on a modest, demographically specific cohort and focused on expressed genomic regions, the findings predicted biologically plausible candidate genes and pathways for further investigation. Future studies would incorporate DNA-based validation, larger ancestrally balanced cohorts, and functional follow-up (such as iPSC-derived neurons or glia edited via CRISPR/Cas9) to confirm and extend these observations.

Several limitations should be considered when interpreting these findings. First, variant discovery from RNA-seq data carries four interrelated limitations: (i) detection is restricted to expressed exonic regions, so variants in unexpressed, lowly expressed, or non-coding regions are not detectable; (ii) RNA-seq cannot reliably distinguish germline from somatic variants without DNA-level confirmation; (iii) RNA-seq is susceptible to RNA editing artifacts, and although we filtered A>G substitutions at known editing sites, novel or rare editing events may not be fully excluded; and (iv) absence of a variant call in non-users may reflect insufficient coverage or low transcript expression rather than true genomic absence, a concern particularly relevant given our cohort-concordance filter. We therefore describe these variants as RNA-seq–detected expressed exonic variants (RDEVs) rather than verified germline susceptibility variants, and DNA-based validation (whole-genome or whole-exome sequencing) in an independent cohort is required for confirmation. Second, the cohort was matched on age and sex but not on self-reported race (13 White/7 Black non-users vs. 19 White/1 Black OUD). Race was included as a covariate in differential expression models, but the small number of non-White subjects limits ancestry-stratified inference; validation in ancestrally balanced cohorts is required. Third, bulk RNA-seq cannot distinguish whether differential gene expression reflects per-cell transcriptional changes or shifts in cell-type proportions. This is particularly relevant for the immune-pathway enrichment reported here, given documented microglial proportion changes in opioid-exposed postmortem brain, and single-nucleus RNA-seq follow-up is required to disentangle these contributions. Fourth, the modest cohort size (*n* = 20 per group) would be substantially underpowered for formal variant-level association testing, which is why the cohort-concordance filter is applied here as a candidate-identification approach rather than as a statistical association test. The same modest size also limits power for DEG detection, particularly for genes with smaller effect sizes. Fifth, label permutation is sometimes proposed for assigning empirical significance to cohort-concordance filters; however, permutation requires sample exchangeability, an assumption that may not hold in RNA-seq data where transcript coverage is itself condition-dependent, as evidenced by the 174 differentially expressed genes identified here. A permutation-derived null would conflate coverage-driven detection asymmetry with random noise rather than producing cleaner inference. Moreover, because a concordance filter can recover qualifying variants even under randomly permuted labels, such recovery would confirm rather than undermine our central caveat: perfect case-control partitioning of RNA-seq variant calls is not in itself evidence of association, which is precisely why we present these variants only as candidates for DNA-based validation. We therefore rely on the analytical bound described in Methods (Section 2.4) and on explicit AF-based caveats for individual candidates, rather than on permutation-based inference. Sixth, several candidate RDEVs have high reported population allele frequencies (AF > 0.8). For these variants, apparent absence in non-users could reflect either true cohort-specific presence or RNA-seq detection limitations; the biological vs. technical origin of this pattern remains an open question that DNA-based validation in matched, ancestrally balanced cohorts will resolve. This validation is identified as the planned next phase of this work. Seventh, all findings are hypothesis-generating, and establishing any candidate as a genuine OUD-associated variant requires a defined evidence chain: (i) DNA-based confirmation of the variant genotype (whole-genome or WES) in the same individuals, to distinguish true genomic variants from RNA-seq detection artifacts and to establish germline status; (ii) genotyping in an independent, adequately powered, ancestrally balanced case-control cohort to test for allelic enrichment in OUD within a formal association framework; and (iii) functional validation in appropriate neural models, such as iPSC-derived neurons or glia carrying the candidate variant introduced via CRISPR/Cas9, to establish mechanistic links between the variant, its transcriptomic consequences, and OUD-relevant phenotypes.

## Data Availability

The original contributions presented in the study are included in the article/Supplementary material, further inquiries can be directed to the corresponding author.

## Supplementary material

Supplementary Figure 1 Top-ranked pathways among downregulated differentially expressed genes (DEGs), ordered by enrichment ratio. None of the displayed pathways reached FDR < 0.05.

Supplementary Figure 2 Predicted long non-coding RNA-messenger RNA (lncRNA-mRNA) interactions and clustering. **(A, B)** Predicted interactions between long intergenic non-coding RNA 01554 (LINC01554) and neuronal PAS domain protein 4 (NPAS4) using Long Non-coding RNA-RNA Interaction Search (LncRRIsearch). **(C, D)** Predicted interactions between LINC01554 and NPAS4 using BiBiServ-RNAHybrid. **(E, F)** Predicted interactions between long intergenic non-coding RNA 00996 (LINC00996) and growth arrest and DNA-damage-inducible beta (GADD45B) using LncRRIsearch. **(G, H)** Predicted interactions between LINC00996 and GADD45B using BiBiServ-RNAHybrid.

Supplementary Table 1 Identification of genetic variants in Opioid Use Disorder (OUD) cohort.

Supplementary Table 2 Identification of differentially expressed genes (DEGs) in the Opioid Use Disorder (OUD) cohort.

Supplementary Figure 3 lncRNA - mRNA network: black = hubs (LINC01554, LINC02605); red = LINC01554 targets (DLX4, ZFP36); blue = LINC02605 targets (GPR4, EMP1, CSF3, IL4R, SLC2A5, SLC11A1, SERPINA3, MYO1G, MAFF, KCNJ15); gray edges = predicted links.
