# Supplementary material for: Deciphering the impact of genetic variants on vulnerability to Opioid Use Disorder
Source: Front Cell Neurosci. 2026 Sep 10;20:1814602. doi: 10.3389/fncel.2026.1814602 (PMC13600877; doi:10.3389/fncel.2026.1814602)
Supplement: Supplementary file 1 [file Data_Sheet_1.pdf]

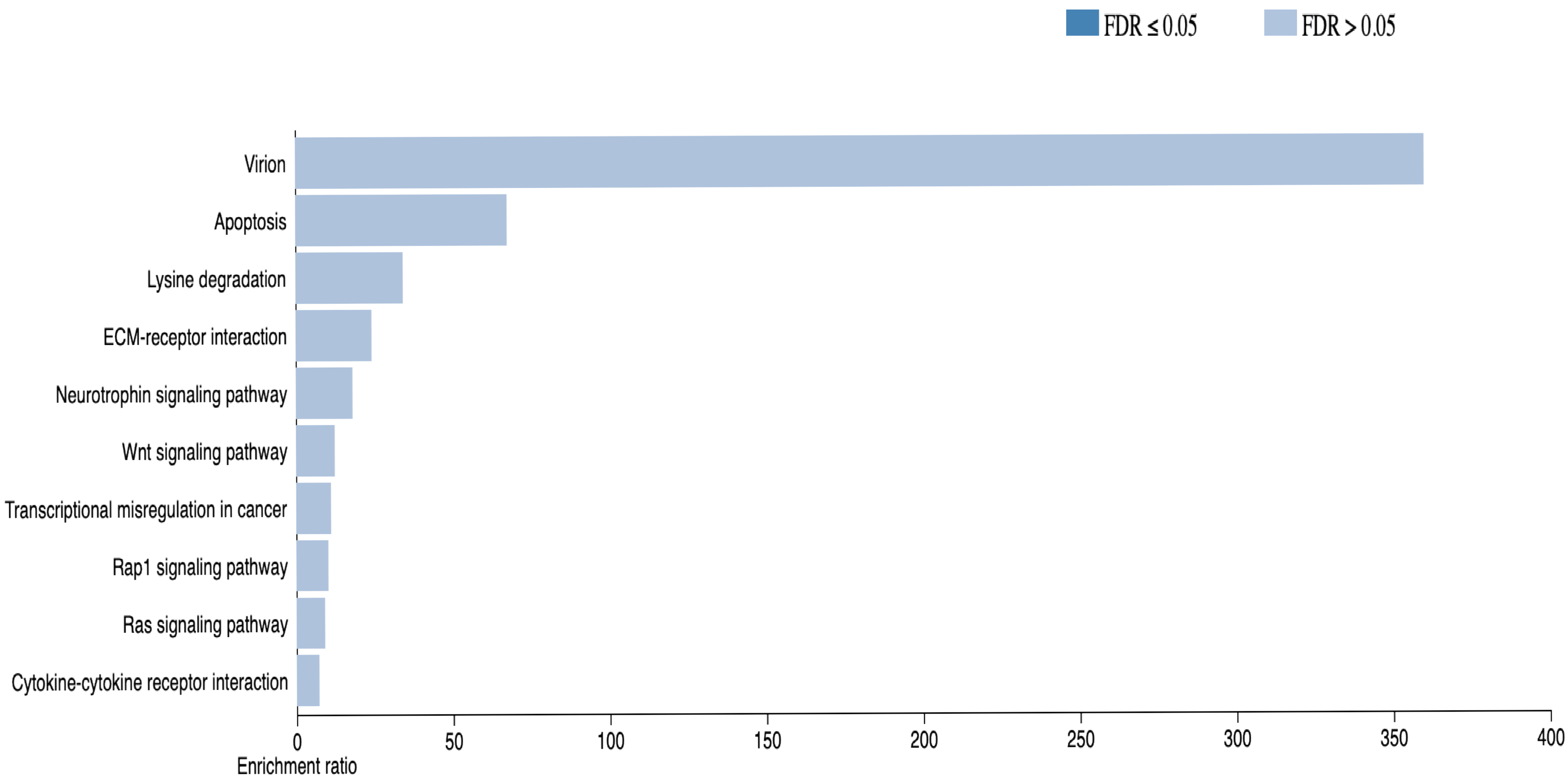
FDR ≤ 0.05
FDR > 0.05
Virion
Apoptosis
Lysine degradation
ECM-receptor interaction
Neurotrophin signaling pathway
Wnt signaling pathway
Transcriptional misregulation in cancer
Rap1 signaling pathway
Ras signaling pathway
Cytokine-cytokine receptor interaction
0
50
100
150
200
250
300
350
400
Enrichment ratio
