# Supplementary material for: Deciphering the impact of genetic variants on vulnerability to Opioid Use Disorder
Source: Front Cell Neurosci. 2026 Sep 10;20:1814602. doi: 10.3389/fncel.2026.1814602 (PMC13600877; doi:10.3389/fncel.2026.1814602)
Supplement: Supplementary file 2 [file Data_Sheet_2.pdf]

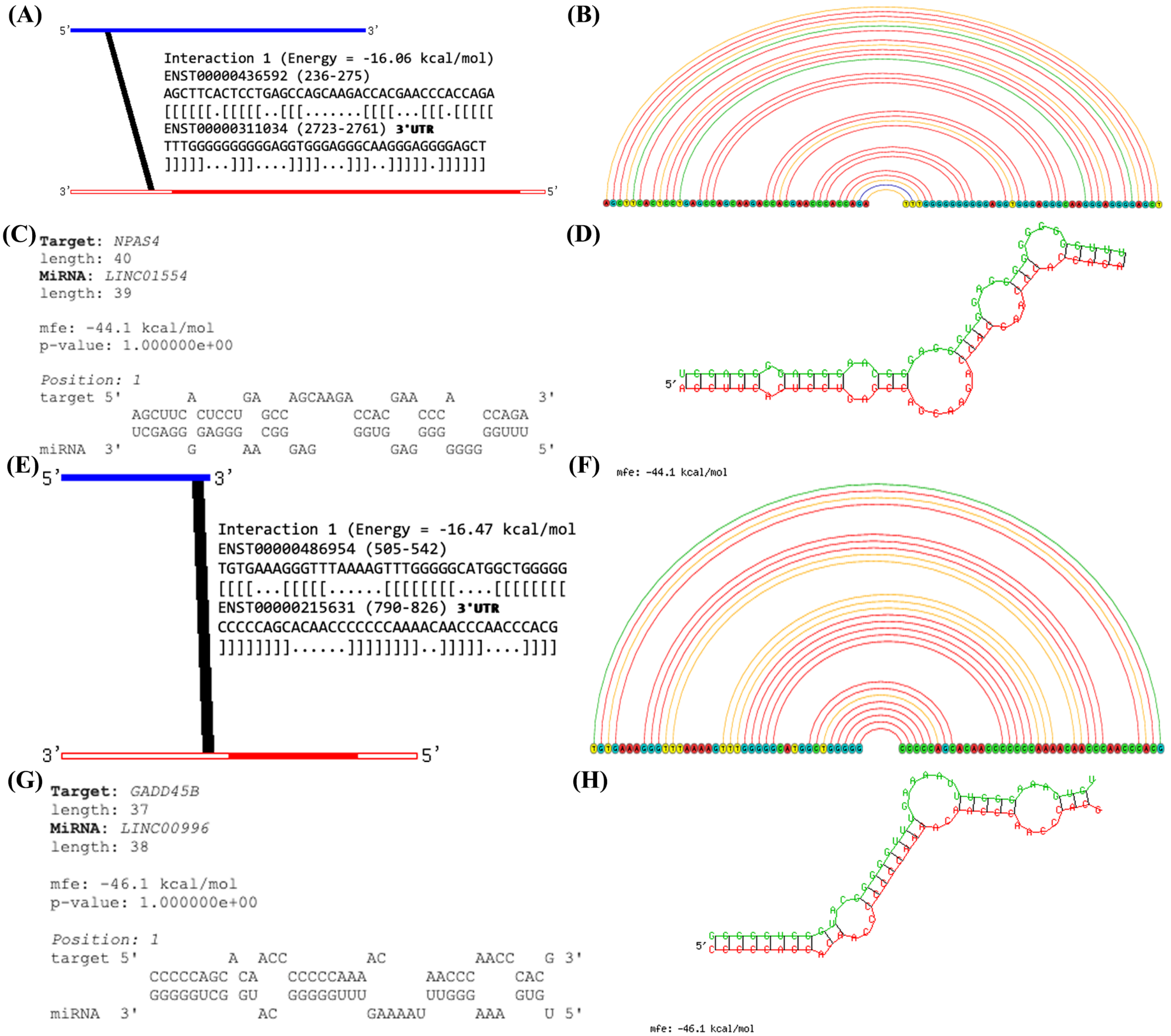
(A)
5'
3'
Interaction 1 (Energy = -16.06 kcal/mol)
ENST00000436592 (236-275)
AGCTTCACTCCTGAGCCAGCAAGACCACGAACCCACCAGA
[[[[[[.[[[[[..[[[.......[[[[...[[[.[[[[[
ENST00000311034 (2723-2761) 3'UTR
TTTGGGGGGGGGGAGGTGGGAGGGCAAGGGAGGGGAGCT
]]]]]...]]]....]]]]...]]]..]]]]].]]]]]]
3'
5'
(B)
(C)
Target: NPAS4
length: 40
MiRNA: LINC01554
length: 39
mfe: -44.1 kcal/mol
p-value: 1.000000e+00
Position: 1
target 5' A GA AGCAAGA GAA A 3'
AGCUUC CUCCU GCC CCAC CCC CCAGA
UCGAGG GAGGG CGG GGUG GGG GGUUU
miRNA 3' G AA GAG GAG GGGG 5'
(D)
5'
mfe: -44.1 kcal/mol
(E)
5'
3'
Interaction 1 (Energy = -16.47 kcal/mol
ENST00000486954 (505-542)
TGTGAAAGGGTTTAAAAGTTTGGGGGCATGGCTGGGGG
[[[[...[[[[[......[[[[[[[[....[[[[[[[[
ENST00000215631 (790-826) 3'UTR
CCCCCAGCACAACCCCCCCAAAACAACCCAACCCACG
]]]]]]]]......]]]]]]]]..]]]]]....]]]]
3'
5'
(F)
(G)
Target: GADD45B
length: 37
MiRNA: LINC00996
length: 38
mfe: -46.1 kcal/mol
p-value: 1.000000e+00
Position: 1
target 5' A ACC AC AACC G 3'
CCCCCAGC CA CCCCCAAA AACCC CAC
GGGGGUCG GU GGGGGUUU UUGGG GUG
miRNA 3' AC GAAAAU AAA U 5'
(H)
5'
mfe: -46.1 kcal/mol
