# Supplementary material for: Deciphering the impact of genetic variants on vulnerability to Opioid Use Disorder
Source: Front Cell Neurosci. 2026 Sep 10;20:1814602. doi: 10.3389/fncel.2026.1814602 (PMC13600877; doi:10.3389/fncel.2026.1814602)
Supplement: Supplementary file 3 [file Data_Sheet_3.pdf]

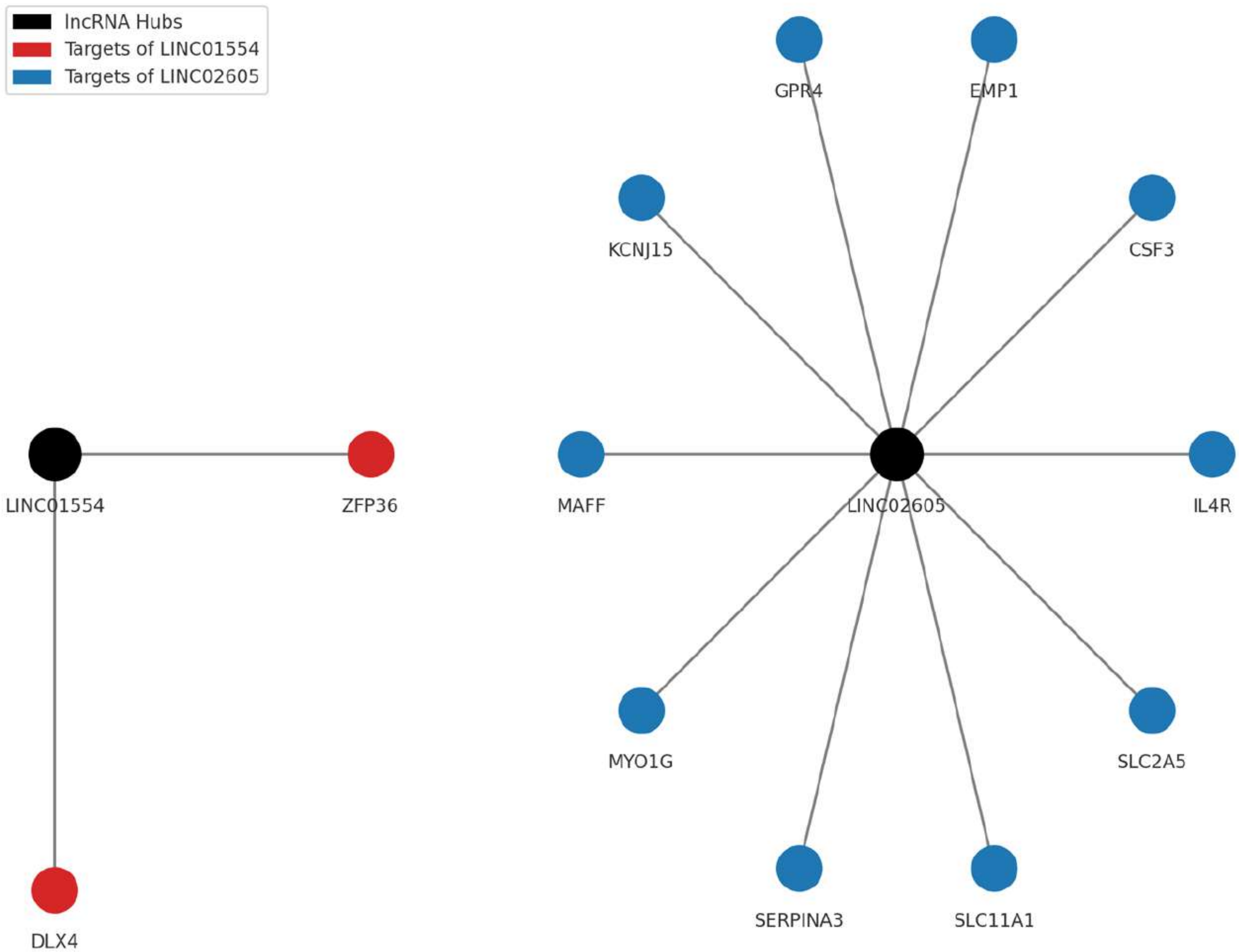

lncRNA Hubs
Targets of LINC01554
Targets of LINC02605
GPR4
EMP1
KCNJ15
CSF3
LINC01554
ZFP36
MAFF
LINC02605
IL4R
MYO1G
SLC2A5
DLX4
SERPINA3
SLC11A1
